# Women's experiences of age‐related discontinuation from mammography screening: A qualitative interview study

**DOI:** 10.1111/hex.13723

**Published:** 2023-02-17

**Authors:** Emma G. Gram, Sigrid W. Knudsen, John Brandt Brodersen, Alexandra Brandt R. Jønsson

**Affiliations:** ^1^ Center for General Practice, Department of Public Health University of Copenhagen Copenhagen Denmark; ^2^ Primary Health Care Research Unit Region Zealand Denmark; ^3^ Department of People and Technology Roskilde University Roskilde Denmark; ^4^ The Research Unit for General Practice, Department of Social Medicine University of Tromsø Tromsø Norway

**Keywords:** age discrimination, ageism, breast cancer, in‐depth interview, Mammography screening, older women, screening discontinuation

## Abstract

**Introduction:**

In Denmark, women are discontinued from mammography screening at age 69 due to decreased likelihood of benefits and increased likelihood of harm. The risk of harm increases with age and includes false positives, overdiagnosis and overtreatment. In a questionnaire survey, 24 women expressed unsolicited concerns about being discontinued from mammography screening due to age. This calls for further investigation of experiences related to discontinuation from screening.

**Methods:**

We invited the women, who had left comments on the questionnaire, to participate in in‐depth interviews with the purpose to explore their reactions, preferences, and conceptions about mammography screening and discontinuation. The interviews lasted 1–4 h and were followed up with a telephone interview 2 weeks after the initial interview.

**Results:**

The women had high expectations of the benefits of mammography screening and felt that participation was a moral obligation. Following that, they perceived the screening discontinuation as a result of societal age discrimination and consequently felt devalued. Further, the women perceived the discontinuation as a health threat, felt more susceptible to late diagnosis and death, and therefore sought out new ways to control their risk of breast cancer.

**Conclusion:**

Our findings indicate that the age‐related discontinuation from mammography screening might be of more importance than previously assumed. This study raises important questions about screening ethics, and we encourage research to explore this in other settings.

**Patient and Public Contribution:**

This study was conducted as a result of the women's unsolicited concerns about being discontinued from screening. This particular group contributed to the study with their own statements, interpretations and perspectives on the discontinuation of screening, and the initial analysis of data was discussed with the women during follow‐up interviews.

## 1 INTRODUCTION

Globally, breast cancer is the second‐most frequently diagnosed cancer and the fifth‐most deadly.^1^ Breast cancer screening aims at detecting localized stages of breast cancer to initiate early treatment and lower morbidity and mortality.^2^ In Denmark, women aged 50–69 are systematically invited to biennial breast cancer screening with mammography.^3^

As the incidence and mortality of breast cancer increase exponentially with age, it may intuitively seem attractive to invite women older than 69 years.^2^ However, participation in mammography screening can, as with other cancer‐screening programmes, unintendedly cause the participants' harm, for example, overdiagnosis, overtreatment and false‐positive tests.^4^, ^5^, ^6^, ^7^, ^8^, ^9^, ^10^, ^11^ The risk of being harmed is influenced by several factors, including the age and health status of the screening participant. Older women with limited life expectancy and competing health concerns have a higher risk of experiencing harm from screening.^12^, ^13^, ^14^, ^15^, ^16^, ^17^ Compared to women screened biennially from age 50 to 69, screening 1000 women until age 74 prevents one breast cancer death, while 78 more women will receive a false positive test result, and additional 8 women will be overdiagnosed and overtreated.^14^ The harms are doubled if biennial mammography is continued until age 80, while only one additional breast cancer death is avoided.^12^ Furthermore, breast cancer detected in older women is more likely to be slow growing and have favourable biologic characteristics, which contribute to the decreased likelihood of benefit from screening and increased likelihood of harm.^12^, ^18^, ^19^, ^20^ Hence, in Denmark, women are discontinued from screening at age 69 due to increased likelihood of harm and decreased likelihood of benefit.

However, most older women are resistant to discontinuing breast cancer screening despite severe health conditions, short life expectancy, information about harm, and physician's recommendation not to be screened.^21^, ^22^, ^23^, ^24^ A survey quantified the prevalence of overscreening and showed that about half of older American adults reported being screened for cancer beyond the upper age limit recommended by the US Preventive Task Force.^25^ This magnitude of overscreening might be explained by the fact that women generally have high expectations of mammography screening, overestimate benefits, and feel reassured of their well‐being from participation.^26^, ^27^, ^28^, ^29^, ^30^ Recent research found that older women appreciate the invitation to mammography screening and interpret it as a symbol that society cares about them.^31^ Combined, these studies indicate that participation in mammography screening is of great importance to women and their psychosocial health.

In line with this, an unexpected finding from a survey suggested that Danish women are unaware of this aforementioned harm‐and‐benefit balance and thus experience the discontinuation from mammography screening as negative. In a follow‐up survey on psychosocial consequences of mammography screening, 24 women returned the paper questionnaire with unsolicited comments outside the template.^32^ In these comments, the women expressed concerns associated with no longer being invited to the national mammography‐screening programme due to their age. These comments substantiated that the discontinuation could cause negative psychological or emotional consequences for the individual woman. One example was a 69‐year‐old woman who just had participated in her last screening round: ‘I wish to continue mammography screening until I turn 80 years old, and not stop participating when I turn 70. It makes me incredibly insecure about my future’ (Jaqueline, age 69). To the extent of our knowledge, no study has previously examined the psychosocial or emotional consequences of age‐related discontinuation from screening. Therefore, this article reports women's experiences, perceptions, and reactions to the age‐related discontinuation of mammography screening.

## 2 METHODS

We conducted a qualitative study with semistructured, individual, in‐depth interviews with 14 participants selected based on free‐text responses to a survey on the psychosocial consequences of mammography screening.

### 2.1 Study design

In 2005, the condition‐specific questionnaire Consequences of Screening—Breast cancer (COS‐BC) was developed and validated to measure the potential psychosocial consequences of false positives in mammography screening.^33^, ^34^, ^35^ The COS‐BC was used in a longitudinal survey following more than 1300 women for 3 years after their participation in mammography screening.^6^ Recently, we conducted a 12–14‐year follow‐up of this study, where 24 women left unsolicited comments about their discontinuation (Appendix A for all 24 written comments on COS‐BC 2019).^32^

### 2.2 Informant recruitment and characteristics

All 24 women, who had written personal comments on COS‐BC, were invited to participate in this study. The women were invited by postal invitations and 14 were accepted. We estimate that 14 informants are sufficient to attain high information power and generate new knowledge.^36^ Data saturation was reached after 8 interviews yet we carried out all 14 planned interviews to ensure that all participants were included in our analysis.

### 2.3 Individual in‐depth interviews

Interviews were held with a focus on the women's narratives, guided by a semistructured interview guide with open‐ended questions (Appendix B).^37^ The interviews were scheduled to last for 1 h, but many women asked interviewers if they could stay longer, as they did not feel 1 h was sufficient time. As a result, interviews lasted 1–4 h. Interviews were conducted where the women found it most comfortable: Nine in the home of the informant, three in our university office and two over the phone. All interviews were audio‐recorded and transcribed verbatim. Interviews were conducted in Danish and translated by the interviewers in collaboration with a native‐English speaker. After the interviews, several women contacted us to elaborate on their thoughts and experiences. On that basis, we decided to contact all by telephone approximately 2 weeks after the initial interview. We asked open‐ended questions about their experience with the interview and asked specifically if they had had any reflections on the subject since. In the end, we presented the women with our interpretation of the first interview and asked for validation. The follow‐up interviews lasted 15–40 min. To ensure that the interviews did not leave the women with worries regarding their health they were given the opportunity to speak to a professor in general practice (J. B. B.), but none felt the need for this. Additionally, in our interview guide, we incorporated an item to inform the women about the medical rationale behind the discontinuation. This was done to prevent imputing any health‐related worries about being discontinued. The women, instead, stressed that they found it pleasant to be able to voice their concerns regarding the discontinuation. The interviews were conducted by E. G. G. and S. W. K. and supervised by A. B. R. J.

### 2.4 Analytic approach

We analysed the data using open, inductive coding, which allowed us to determine common themes and patterns across the women's experiences. E. G. G. and S. W. K. coded together and discussed codes until a consensus was reached. We employed thematic network analysis to systematize the complex web of interpretations and themes.^38^ We chose this analysis approach, as it allows us to explore patterns across interviews. We continuously revisited themes and transcriptions to systematically verify and validate the interpretations of data. All authors read transcripts and discussed interpretation and analysis. Initial draft was written by E. G. G. and A. B. R. J. and developed in close collaboration with the two other authors.

## 3 RESULTS

The women were invited by postal invitations. All informants are presented by pseudonyms, and identifiers have been altered to secure anonymity. Socioeconomic and demographic characteristics are presented in Table 1.

**Table 1:** Demographic characteristics of informants.

Pseudonym	Age	Residence	Occupation	Self‐reported screening results
Susan	67	Urban	Retired social worker	True positive
Sophia	71	Urban	Public worker in art institution	True negative
Lily	66	Rural	Retired service and administrative worker	False negative
Mia	75	Urban	Retired social worker	False positive (also in pap‐smear)
Amelia	72	Rural	Retired social worker	True negative
Freya	73	Rural	Retired health worker	True positive
Elisabeth	71	Rural	Artist. Retired social worker	False positive (had a lumpectomy before the lump was acknowledged benign)
Linda	81	Urban	Unknown. Retired	True negative
Jaqueline	78	Urban	Retired health worker	True negative
Barbara	72	Urban	Retired health worker	True negative
Patricia	74	Urban	Retired civil servant	True positive
Nancy	73	Urban	Retired administrative worker	True negative
Betty	72	Urban	Retired social worker	False positive (also in pap‐smear)
Margaret	73	Rural	Artist	False positive

In the analysis of the interviews, four main themes emerged; first, that screening was regarded as a positive means to help improve public health and thus, one had a moral obligation to participate. Second, the screening discontinuation was considered a societal degradation and the women felt subject to ageism. Third, the possible harms of screening for the individual were judged inferior to the benefits of the common good. Finally, screening was used to control one's health, and lack of screening would make women more susceptible to cancer and death.

### 3.1 Participating in screening is a moral obligation

The women in this study perceived screening as a positive offer to them and society. For instance, in more informal conversations, several women credited the invitation to screening as ‘…very nice of them’, referring to health authorities. Following this, the women believed that participation in screening should be accepted:

I think that when you're offered screening, then you should take it. Most certainly. A lot of people don't want to. I don't get that. We should be pleased that we are offered screening. (Barbara, 72)

This quote emphasizes that participation in screening ought to be accepted simply because the authorities offer it. Women generally base these statements on arguments that screening must be good otherwise the authorities would not offer it. But, participating in screening was seen not only as something to do for one's own sake, yet also as a duty; something that was for the good of society since preventing disease could save the government money:

Well, for me. I've always attended to avoid breast cancer. That's obvious, right? I'm aware that screening prevents it and fewer people will get it (breast cancer) and thereby we can save money and so on. (Elisabeth, 71)

It also came up during the interviews, that there was a morality implicit in participating. Not just in terms of saving money, but also that participating in screening was the right thing to do; according to the women in the study, other women who chose not to participate in screening programmes were not just harming themselves but were acting morally wrong because it was seen as an act of choosing one's own ideals over the greater good of the society. The women described an obligation to stay healthy and take care of their health, for example, by participating in screening. The women applied condescending terms, such as ‘lazy’, to themselves when they felt that they had not done enough to protect their health. They expressed feelings of guilt, for example, when they did not examine their breasts between screenings. The women were, contrarily, very proud to participate in screening:

…(mammography screening) was a good thing, and it was important, and it was something you were very proud to have done. (Sophia, 71)

It also became clear, that participating in screening was like entering a mutual agreement with the state; that you would do your best to take care of your own and others' health by attending the screening, and then in return, the state would treat you if you were to become ill. The women reported high trust in the authorities. When asked how they would react to a recommendation from a doctor that they did not agree with, they all shrugged their shoulders and said that they would simply do as told, because the doctors knew best. Some exemplified by referring to events in which health professionals had secured better treatment or even saved lives, while others recalled situations in which they had felt comforted and praised by the Danish healthcare system.

I trust in them, they know better than I do (…). One has faith in the authorities, right? (Mia, 75)

Or as Susan put it:

They (health professionals) make me feel safe. They know what they are doing. (Susan, 67)

Despite previous negative experiences or encounters with health services, the women still trusted health authorities. One example was Lily, who had contacted her general practitioner (GP) as she suspected breast cancer in her left breast. Her GP told her that nothing was wrong, she just needed to deal with her nerves. Lily was embarrassed even though she still believed something was wrong. After debating with herself, the following year Lily returned to her GP and complained about her left breast again. This time she had a biopsy taken, which showed that she in fact did have breast cancer, and subsequently, her left breast was surgically removed. Lily explicitly did not hold a grudge, and never had, rather, she continued screening because it must be good since it was offered. In sum, the women intuitively participated in screening due to high trust in authorities. Participating in screening was considered a moral obligation because screening is a way to take responsibility and support own health, and also because screening would save state finances.

### 3.2 Screening discontinuation is a societal degradation

None of the women were aware of the medical reasons for the discontinuation. The women had instead made up their own interpretations that older women were considered worthless from a societal perspective and consequently they felt subject to ageism when discontinued. The quote from Margaret illustrates the feeling of age discrimination:

…I think it's degrading that they didn't think women above 69 needs it (mammography screening), it's degrading to say: they're over anyways (…) that's how I felt it! That there's no reason to take care of us (women aged >69) anymore; we cannot give birth anymore, we're not sexually attractive anymore—never mind if we get cancer! (Margaret, 73)

The feeling of being perceived as worthless was further based on the assumption that older women did not contribute to society, for example, due to retirement. Still being able to work was frequently offered as proof of societal value:

I think it's rude to us. Because we are … many of us are still working, actually—if not full time then part time, and we are useful citizens! (Sophia, 71)

The women's general understanding was that society needs to make financial sacrifices in health care and therefore mammography screening for older women was given lower priority, but they still felt subject to age discrimination:

I fully understand that the healthcare system can't test everything and everyone into eternity. I know that (…) but personally I was insulted. To be written off. To be deemed out. (Elisabeth, 71)

The prioritizing, however, was understood by the women as now that they had become older, they were not of much use to society. The women told that instead of rewarding them for the good they had done, for performing morally well as citizens by attending screenings and taking care of their health, society had disregarded them. In sum, being discontinued owing to age was a degradation of your worth as citizens and thus an expression of ageism. However, it had a positive effect on their feeling of age discrimination when informed about the medical rationale for discontinuation.

### 3.3 Harms of screening are not important

We informed the women that the harms of mammography screening increased with age and that the benefits decreased. The women did not question the harms we presented them but generally did not care. Rather, the interviews revealed that the women, as also stated in the previous section, preferred to continue screening. They were asked directly to tell their thoughts on the balance of harms and benefits, and this showed, that all the women inferred that the benefits outweighed the harms no matter how large the harms were. Some of the women related mammography screening to vaccination programmes and stated that they are for the greater good, though some might experience unintended harm. The women generally referred to the principle of ‘better safe than sorry’ and some expressed that they preferred overdiagnosis ahead of the possibility of some cancers not being detected:

…I would say, better treat one time too much. It could be a cell which was not cancerous, it was just a normal benign one (…) rather one time too much, than one time too little. (Amelia, 72)

Four women previously had false positive‐test results, but incessantly gave positive statements about screening. They further agreed that even though some women might suffer from negative consequences of screening, continuous participation was far more important as lives could be saved. Finally, the women attached little importance to the psychosocial consequences following false‐positive findings and expressed high tolerance towards this unintended harm:

Well … you will just have to try to live with it. I simply feel like saying: move on, the sun is shining! Of course, I could be struck in the head by a cobble, but the risk is not going to make me afraid. (Nancy, 73)

In fact, one woman commented, that psychological harm should be disregarded for the sake of the benefits:

…they will just have to accept those psychological things, I will say, better that, than the opposite (false negative). (Sophia, 71)

To sum up, the women in this study found that benefits outweighed harms; potential harms for the individual were judged inferior to the benefits of the common good. In addition, the women reported high tolerance towards the harms of breast cancer screening. This led to the judgement that the women would like to continue screening beyond 69 years.

### 3.4 Striving for new means of control

The favourable judgement for continuing mammography screening in older women was rooted in high expectations to screening. For example, the women believed that participation saves lives. This was repeated in all interviews, and many did not only refer to benefits concerning their own life but also that of others:

We just need to participate in screening, so we can save them all—that's what this is about! (Linda, 81)

Now (with screening) we could save TONS of women from getting breast cancer! (Mary, 80)

Also, screening was expected to detect any asymptomatic disease that they were not able to themselves:

…it (screening) can detect a lump before you can detect it yourself, it's actually quite simple! There's a great chance of finding something at a screening and thereby avoiding death. (Sophia, 71)

All the women in this study said they felt reassured that they were in fact not ill when participating in screening. Nancy exemplified how screening was viewed as a technique by which you could obtain bodily control and monitor own health:

I think I would say that screening is an insurance, it is something that supports health or prevents accidents from getting awful! It is a kind of ‘safety first’ so we can get ahead of it, if it were to come. (Nancy, 73)

Susan gained reassurance through mammography screening, which relieved her from some of the worries she had concerning staying healthy and beware of bodily symptoms, and so forth:

I have been extremely pleased by the fact that someone takes care of it for you. It was like a burden taken off my shoulders. (Susan, 67)

Risk was generally considered an inescapable part of life. The women, however, accentuated that the risk of breast cancer could be controlled by participation in screening. Overall, the women believed participating in screening was a mean to avoid illness and potential death, and that discontinuation would, therefore, have a negative effect on their health and life expectancy:

…if you quit at 70 years (screening), and I count on turning 103, and then if I get more trouble with breast cancer, and I can't check myself, then it's nice that I can get the screening and receive a check‐up. (Freya, 73)

The women believed that the lack of screening would also make them more susceptible to late breast cancer diagnosis and death:

It makes sense to screen because we detect so much that we wouldn't have detected if we didn't screen (breast cancer). When we are not offered screening, it will be detected too late and thereby we cannot save them…. (Nancy, 73)

Believing that screening saves lives, the women strove for new means to obtain control over their health after screening discontinuation. On their own initiative, the women pointed out that they ought to discuss further examinations of their breasts with a GP. Some women had already made arrangements with their GP, assuring that they could be referred to a mammography, if they had the least of worries. Others had paid for mammograms in the private health sector. To sum up, the women had high expectations of the benefits of screening and therefore felt more vulnerable to breast cancer, disease and death when they discontinued mammography screening.

## 4 DISCUSSION

The 14 interviews revealed that the women considered attending mammography screening as a moral obligation that accommodates private health benefits and financial benefits for society. Being discontinued from screening, therefore, was experienced as age discrimination and was believed to be due to older women being given less societal priority. When women were informed about the increasing harms and decreasing benefits of continued screening, they responded that harms did not matter and even a slight mortality benefit would outweigh any degree of harm. The women argued pro screening due to high expectation to screening including that it saves lives and thereby allows for controlling your health. Therefore, when discontinued from screening, the women felt more susceptible to breast cancer, late diagnosis, and death. Consequently, the women strove for new means of controlling their risk of breast cancer, for example, by opportunistic screening.

### 4.1 Findings in a Danish context

Denmark, a Nordic welfare state, has a publicly funded healthcare system with free and equal access to services. All citizens in Denmark contribute to the healthcare system through taxation, and everyone funds the costs for national screening programmes, whether they participate or not. The Nordic welfare state entails an implicit understanding that society is to benefit all, and that each individual is responsible for the welfare by paying taxes and following rules of conduct. This includes taking care of one's health to avoid putting strains on the system finances.^39^, ^40^ This might explain why the women place great emphasis on participating in screening and feel responsible for maintaining good health.

### 4.2 Comparison of findings

The women believed and argued that screening saves lives. This is also the case in previous literature, which shows that people generally have strong, positive beliefs about the benefits of cancer screening.^28^, ^30^, ^41^, ^42^, ^43^, ^44^ Literature also supports that lay people generally overestimate the benefits of participation.^23^, ^26^, ^27^, ^45^ A Danish study examined responses to mammography screening and revealed that it provided feelings of reassurance for those participating, compared with women not invited to screening.^30^ Another Danish interview study also found that women with false‐positive results did not blame medical technology or lose confidence in screening but continued to desire participation despite their negative experiences.^46^ Robert Crawford^47^, ^48^ introduced the theory of Healthism and emphasized that control and anxiety are connected in a spiral relation, where one enhances the power of the other. When control is obtained through participation in mammography screening, the more apparent it becomes that the risk of breast cancer needs to be controlled. Crawford further explained that individuals develop a need to gain control over risk factors to feel secure due to increased awareness and attention to risk factors and personal responsibility. The women became aware of their risk of breast cancer through mammography screening, and this awareness generated feelings of anxiety and insecurity, which in return motivated further attempts to control risk. This neoliberal development where individuals increasingly are considered responsible for their own health is also apparent in our findings. In other settings, women who have had false‐positive experiences in mammography screening and thereby the risk of breast cancer close to their attention were found to continuously seek medical confirmation of their well‐being and desire more screening.^46^, ^49^ This underpins how attention to risks enhances the need for controlling these risks. An Australian study examined reactions to less extensive cervical‐cancer screening following the renewal of the Australian cervical‐cancer screening programme, which lowered frequencies of screening.^50^ The study revealed that women who have had personal experiences with prestages of cancer or cancer through themselves, family or friends felt particularly vulnerable to the renewal. They were convinced to be at higher risk of late diagnosis, and death due to cervical cancer, felt a stronger appreciation for the former screening programme, and assumed that it had prevented their death or that of a friend or family member. This is in accordance with our findings, where women feel more susceptible to breast cancer after discontinuation and seek out alternative ways of control, for example, by making arrangements with their GP to make sure that they, in their own perception, were properly checked for breast cancer. This tendency is unlikely to be limited to a Danish setting, despite the free healthcare access. An American study shows that 74.1% of American women older than 74 years undergo breast cancer screening despite the US Preventive Task Force recommendation not to.^25^ Previous work addressing the communication of discontinuing mammography screening supported that women feel devalued when discontinued from screening,^23^, ^51^, ^52^ and perceived inviting older women to screen as a symbol that society still cares about them.^31^ The participants in our study intuitively assumed that age‐related discontinuation from screening was a case of ageism. From the women's statements, we interpreted that they do not think that the general representation of older women in breast cancer screening amplified any predictive potential. In other words, the women did not think that societal stereotypes of older women were anywhere near the truth. For example, the women experienced themselves as being subject to stereotyping, where they all resembled old women who were not working or not contributing to society by any means. This also supports our overall interpretation that screening is not only a preventive tool but bears a larger societal and cultural meaning for the women invited. In Denmark, there is no official information material given on why women are discontinued from screening at age 69. This was also reflected in our interviews as the women were not aware of official reasons or medical rationale to stop breast cancer screening. Recently, regions of Australia and Canada have been some of the first to introduce information on this matter.^24^, ^53^ This might ease the women's feeling of age discrimination, as it did in our interviews. However, studies examining older adult's decision‐making in cancer screening, reveal that older adults continue to desire participation despite information about harms, increased likelihood of harms, and reduced likelihood of benefit.^22^, ^24^, ^31^, ^41^, ^54^ This indicates that emotions and intuition play a role in screening decisions rather than evidence about potential benefits and harms.^23^, ^41^, ^54^, ^55^ The literature addresses this discrepancy between evidence and women's understanding of the information as a perception gap and suggests that it makes women unable to properly consider discontinuing screening.^24^, ^45^, ^55^, ^56^ This hints that the decision to attend screening is more complex than the balancing of benefits and harms, while it also explains why the women in this study disregarded the importance of harm in screening decisions.

### 4.3 Strengths and limitations

The individual semistructured interview was a cogent method for this study as it explores individual thinking, emotions and personal stories. We conducted follow‐up interviews that allowed additional depth since the women had time to reflect on the topic and the chance to further elaborate. The women were generally happy to talk to us and left the interview in a good state. We invested time in making the women feel comfortable and listening to their unrelated worries or stories, which was the primary cause of the long length of the interviews. We assume that women, who left comments on COS‐BC, represent the ones who felt the strongest negative emotions towards the screening discontinuation. It is possible that the women who choose to participate in screening generally trust health authorities more than those not participating. Further, the women who agreed to participate in this interview study are likely to be different from those who did not wish to engage, for example, in regard to socioeconomic status and health. Therefore, the study population might not be representative of all Danish women discontinuing mammography screening. We did not compare these findings to another population to test generalizability. Therefore, the generalizability of findings is limited, and we do not know if these findings are relevant on a larger scale. However, interpreting the findings, we have relied on the women's own interpretations, and conducted follow‐up telephone calls to validate interpretations, so that hopefully no woman felt that her words were taken out of context or overinterpreted.

## 5 IMPLICATIONS

Our analysis points to how some women may experience age‐related discontinuation as a result of neglect or ageism. In this study, the women were selected because they had left unsolicited comments on a questionnaire expressing their concerns about discontinuation. The women associated the discontinuation with being potentially harmful to their health and strove for new means to control general health and the risk of breast cancer. Further, the women explicitly believed the discontinuation to be ageist, and as a result, they felt devalued. We interpret these psychological and emotional reactions as a consequence of mammography screening and age‐related discontinuation. These reactions, propose that age‐related discontinuation from breast cancer screening could be considered a potential unintended harm of the screening programme. New items could be developed as a mean to measure this with quantitative methods. When the women were informed about the medical reasoning for the discontinuation, it mitigated the negative consequences. This suggests the need to develop evidence‐based lay information material on mammography‐screening discontinuation. Previous studies advocate that women tend to evaluate screening behaviour on the merits of emotions, rather than evidence about potential benefits and harms. Therefore, we suggest that future research explore alternative methods of education and effective communication preferably in co‐creation with older women. Generally, the evidence about the experience of being discontinued from mammography screening is sparse and should be investigated in other populations and settings.

## AUTHOR CONTRIBUTIONS

**Emma G. Gram**: Design; interviews; transcription; analysis; writer of the first draft. **Sigrid W. Knudsen**: Design; interviews; transcription; analysis; manuscript revisions. **John Brandt Brodersen**: Design; manuscript revisions. **Alexandra Brandt R. Jønsson**: Design; analysis; supervision; manuscript revisions.

## CONFLICT OF INTEREST STATEMENT

The authors declare no conflict of interest.

## ETHICS STATEMENT

We have followed Social Research Association's guidelines throughout this study. Independent ethical approval was not necessary as The Danish Research Ethical System does not require ethical approval of qualitative interview studies. The survey of COS‐BC provided data for recruitment and was approved by the Danish Data Protection Agency, 2007‐41‐0777. All women had provided written consent to be contacted by the research team.

## Data Availability

The data are not publicly available due to privacy and ethical restrictions.

## APPENDIX A

(Table A1)

**Table A1:** Written comments on COS‐BC.

Pseudonym	Age	Comment
Lisa	74	‘I was very disappointed to be stereotyped and not be called in for mammography screening. Now suddenly I was seen as being too old’
Amelia	72	‘I just think that it is too bad that once you turn 70 years old, you can't get mammography screening anymore’.
Betty	71	‘I would like to continue to participate in mammography screening, because these examinations make me feel safe. I know other women my age (71 years old) who have had lumps detected in their breasts!’
Margaret	73	‘Why does it stop at age 70? If you are lucky to live 20 years more, it is both concerning and devaluing that screening stops before!’
Jacqueline	78	‘I can't seem to remember when the expenses of mammography screening for older women were cut back’
Charlotte	71	‘As we live and work longer, I find it extremely strange, that we cannot get these screenings anymore’
Elisabeth	71	‘I feel very fortunate to live in a country that offers mammography screening. I am sad to be too old to participate anymore’
Nancy	73	‘I am truly thankful for living in a country that offers preventive health screenings’
Mia	75	‘I really do appreciate the follow‐up examination’
Linda	81	‘I am truly thankful for the examinations. My sister died from breast cancer at age 38 ☹’
Patricia	74	‘The years following the examinations I thought a lot about cancer’
Barbara	72	‘But I would still like to be screened’
Helen	73	‘Why don't I get the screenings anymore?’
Donna	77	‘Unfortunately, it is a long time since I had the screenings’
Sarah	75	‘Why does mammography screening stop?’
Maria	75	‘Even though I do not have cancer right now. I can develop cancer in 2–3 months (!!!) ☹’
Anne	75	‘I have always been pleased with the examinations/control’
Susan	67	‘The examinations/mammography feels like a help. The results though can be a burden!’
Freya	73	‘Mammography is good! Otherwise, my cancer would never have been detected’
Debra	79	‘I had cancer detected in July with metastases in the other breast. I had no symptoms of cancer. I should have been invited to screening after the age of 70’.
Sophia	71	‘The only problem I have with screening is that I am 71 years old and I was told that I am no longer invited to participate in mammography screening. As we live longer, I believe that is its extremely strange that we can't get these screenings. It makes no sense to answer this questionnaire as I, apparently, am of no interest to you anymore, and you do not place interest in screening women's breast after they turn 70 years old’.
Mary	80	‘I am very pleased with the invitation to screening’
Karen	75	‘I will now go to see my own doctor to have a breast cancer screening’
Lily	66	‘I have had breast cancer, despite the fact that I did always follow the screenings’.

Abbreviation: COS‐BC, Consequences of Screening—Breast cancer.

## APPENDIX B INTERVIEW GUIDE

**Briefing**

(1) Presentation of us and the project
(2) Informed consent and rights as an informant
(3) Matching expectations: do not answer anything you do not feel like or makes you uncomfortable. This is to be a friendly conversation.
(4) Questions about the interview?
(5) Thank you for replying, did you understand what we wanted? What made you react?

**About you**

(1) Tell us about yourself. Age, name, everyday life, social relations, and so forth.
(2) How do you perceive your own health and health in general?
(3) Experience with screening results?

**Mammography screening, the comment and the cut‐off** What was your motivation for writing a comment on the questionnaire? Interpretation of mammography as phenomenon and tool

(1) Motivation to participate? thoughts? Did you reflect prior to participating?
(2) Can you tell if the participation had any effect on your everyday life?
(3) Do you have any feelings towards mammography screening? If so, can you tell me about them?
(4) Why do you think that we screen for breast cancer? What is the aim of screening?
(5) Who told you what you know?
(6) In Denmark screening stops at age 70, what do you think about that?

(i) Do you suspect that something will change for you after now being screened? Would you do anything in a different way?
(ii) Will it affect your life or have any consequences?

**Age and risk—How do you understand your own risk relative to breast cancer**

(1) Do you feel vulnerable to breast cancer? What affects your risk? Age? Screening?
(2) Do you have a higher risk of breast cancer when you are in a screening population? If so, how does it feel to be discontinued from screening.

**Abstract thinking** So here at last, I would like to present you to some information about mammography screening. If I tell you that (mentions harms of screening) is the reason why screening has an age limit, what do you think about that? Is it fair? How would you like it to be?

(1) Would you still like to be screened?
(2) Why do you think people still would like to be screened, even when they know about these potential harms?
(3) What of the aforementioned harms did you most care for?

**Debriefing**

(1) Thank you for participating
(2) Questions? Something you'd like to add?
(3) Can we contact you again if we…?
(4) Can I take a picture of X (if something's relevant)?
(5) Last question: if you were to write a project about older women and mammography screening, what would be your focus? What would you ask? If you were to construct your own mammography screening programme, how would it be?
